# Ovarian Toxicity in Female Rats after Oral Administration of Melamine or Melamine and Cyanuric Acid

**DOI:** 10.1371/journal.pone.0149063

**Published:** 2016-02-11

**Authors:** Jiarui Sun, Xinchen Zhang, Yinan Cao, Qiling Zhao, Endong Bao, Yingjun Lv

**Affiliations:** College of Veterinary Medicine, Nanjing Agricultural University, Nanjing, China; Utrecht University, NETHERLANDS

## Abstract

Although the toxicity of melamine to the kidneys and testes is well known, few studies have investigated the effects of melamine on female reproductive organs. Therefore, this study explores the effects of oral administration melamine or melamine and cyanuric acid for 28 days on the ovaries of female rats. Rats that were exposed to the mixture exhibited reduced ovarian and uterine weights, a shorter estrous cycle, and reduced serum estrogen and progesterone levels compared to rats that were exposed to melamine and control rats. Furthermore, morphological analysis revealed pathological changes in the ovaries of rats exposed to melamine or the mixture, such as more atretic follicles and necrosis of oocytes and granulosa cells. TUNEL staining revealed that the exposed groups had a higher proportion of TUNEL-positive granulosa cells than the control group, and the mRNA expressions of *SOD1*, *GPX1*, *GPX2*, *P450scc*, *17β-HSD I*, and *17β-HSD II* were reduced in the exposure groups compared with the control group. These results indicated that exposure to melamine alone or to the melamine-cyanuric acid mixture could damage the ovaries in rats.

## Introduction

Melamine is an organic base that is a cyanamide trimer, and it is composed of a 1,3,5-triazine skeleton. It is widely used for the manufacture of plastics, resins, and fabrics. Previous toxicological studies have demonstrated its low toxicity and that it is excreted within 24 h by the kidney following administration [1–3]. However, in 2008, many children in China were reported to have fallen ill and even died following contamination of milk with melamine which caused by nephrotoxicity associated with the accumulation of melamine-uric acid in the kidneys [4–7]. In addition, cyanuric acid is a by-product during melamine production [8]. Cyanuric acid has been reported to be nontoxic [9–10]. However, melamine-cyanuric acid crystals have been known to damage the kidney. Furthermore, studies on animals have found that cyanuric acid and melamine when present in pet and livestock feed caused kidney damage, including degeneration and necrosis of renal tubule epithelia, proliferation of connective tissue, and acute kidney failure [1, 11]. Furthermore, in 2007, the presence of cyanuric acid and melamine in pet food have reportedly caused the death of thousands of companion animals in the USA [1]. These two incidents generate more concerns about the toxicities of melamine and cyanuric acid.

Thus far, melamine and cyanuric acid have been reported to cause kidney damage as well as testicular lesions. After male mice were exposed to melamine or a mixture of melamine and cyanuric acid, they exhibited reduced testicular and epididymal weights; reduced serum testosterone level; and sperm and germ cell lesions including sloughing, necrosis, and apoptosis [12–14]. Moreover, both melamine and the melamine-cyanuric acid mixture were found to damage the Sertoli cell vimentin and disrupt the blood-testis barrier [15]. Such obvious damages to testis made us consider the effects of melamine and the melamine-cyanuric acid mixture on female reproductive organs. To the best of our knowledge, no studies have investigated this association thus far; however, some studies on the effect of melamine on female rats indicated that melamine might be toxic to the female reproductive system. When pregnant or lactating rats were treated with melamine or the melamine-cyanuric acid mixture, melamine was found in amniotic fluid, breast milk, and fetuses. Moreover, both melamine and the melamine-cyanuric acid mixture affected the fetal development and increased the number of early and late fetal deaths [16–19]. However, none of these studies have investigated the damage that these compounds cause to female reproductive organs. In this study, we investigated ovarian lesions in young female rats after they were exposed to melamine or melamine-cyanuric acid mixture, with the aim of understanding melamine toxicity in greater detail.

## Material and Methods

### 2.1 Ethics statement

All animal procedures were approved by the Institutional Animal Care and Use Committee (IACUC) of Nanjing Agricultural University. The study protocol was reviewed and approved specifically (project number: 31101786). The animal sacrifice and sampling procedures strictly followed the “Guidelines on Ethical Treatment of Experimental Animals” (2006) No. 398 set by the Ministry of Science and Technology, China, and the “Regulation regarding the Management and Treatment of Experimental Animals” (2008) No. 45 set by the Jiangsu Provincial People’s Government.

### 2.2 Chemicals

Melamine (MA, purity > 99%, CAS 108-78-1) and cyanuric acid (CA, purity > 98%, CAS 108-80-5) were obtained from Shanghai ANPEL Laboratory Technologies Inc. (Shanghai, PR China). All the other chemicals used in the study were of analytical grade or higher.

### 2.3 Animals and treatment

Seventy female Sprague-Dawley rats (28 days of age), with an average body weight of 100 ± 20g, were purchased from Laboratory Animal Center of Yangzhou University. The rats were housed in separated plastic cages at a temperature of 22 ± 2°C and humidity of 50%, and fed a standard chow diet and water *ad libitum*. The chow and water were confirmed to be free of melamine and cyanuric acid by gas chromatography/tandem mass spectrometry. After 1 week of adaptation, rats were separated into seven groups (10 rats per group). Of these, three groups received 10, 20, or 40 mg of melamine (MA) alone per kg body weight per day. Another three groups received 10, 20 or 40 mg of melamine and cyanuric acid (MC)/kg body weight per day (10 mg·(kg·d)^–1^ MC: 5 mg·(kg·d)^–1^ melamine + 5 mg·(kg·d)^–1^ cyanuric acid). These compounds were dissolved in corn oil and given once daily by oral gavage in a volume of 10 ml/kg body weight for 28 consecutive days. The control animals received the same volume of corn oil but without MA or CA. All the rats were inspected daily after they were administered MA, MC, and corn oil. The rats were weighed, and then euthanized by CO_2_ inhalation in designated CO_2_ chambers after the final treatment. The ovary and uterus were collected and weighed, and the relative weights of the ovary and uterus were calculated as a proportion of each animal’s body weight. Blood samples were collected from the retro-orbital sinus during the diestrus phase, determined by vaginal smear, and centrifuged at 3000 rpm to obtain serum. Five ovaries and kidneys were fixed in 10% formaldehyde, and the remaining five were stored at –80°C until analysis.

In addition, a preliminary study was performed to confirm the toxicity of cyanuric acid (CA) on the female ovary before the above experiment, although CA has been reported to be nontoxic. Ten female rats were separated into two groups (5 rats per group). One group received 40 mg·(kg·d)^–1^ of CA alone (dissolved in corn oil) for 28 consecutive days. Another group received the same volume of corn oil as the control. All the rats were inspected daily after they were administered CA and corn oil. The ovary and blood samples were collected and stored as described above.

### 2.4 Detection of estrous cycle

The estrous cycle was determined by vaginal smear. Ten days before the end of the experiment, vaginal smears were collected at 8–9 AM every day, and stained with Giemsa solution. The vaginal smears were examined under a light microscope (Olympus, Japan). The proestrus smear consisted of predominantly nucleated cornified cells; the estrus smear consisted primarily of enucleated cornified cells; the metestrus smear consisted of an equal proportion of leukocytes, cornified epithelial cells, and nucleated epithelial cells; and the diestrus smear primarily consisted of leukocytes.

### 2.5 Histopathological analysis

The fixed kidney and ovary samples were dehydrated by an alcohol series, clarified in xylene, and embedded in paraffin. The samples were then serially sliced into 4-μm sections. The stained sections were then examined under a light microscope (Zeiss, Germany).

### 2.6 Number of atretic follicles

Five representative sections were selected from each ovary. The interval between sections was >200 nm to avoid counting follicles twice. The follicles were classified according to the method described in a previous study [16]. Briefly, primordial follicles are defined as oocytes surrounded by one layer of flattened somatic cells; primary follicles, oocytes surrounded by two or three layers of cuboidal granulosa cells without antral space; and antral follicles, oocytes with three or more layers of cuboidal granulosa cells and one or more independent antral spaces. Follicles were determined to be atretic if they displayed two or more of the following criteria within a single cross-section: more than two pyknotic nuclei, granulosa cells within the antral cavity, granulosa cells pulling away from the basement membrane, or uneven granulosa cell layers. The numbers of total and atretic follicles were counted in each treatment group. The index of atretic follicles was calculated as a percentage of all follicles.

### 2.7 Serum estrogen (E_2_) and progesterone (P) levels

To avoid the effects of cyclical variations of the estrous cycle, blood samples were collected during the diestrus phase. The serum E_2_ and P levels were determined on individual samples using special radioimmunoassay kits (Beijing North Institute of Biological Technology, Beijing, China)

### 2.8 Terminal deoxynucleotidyl tranferase-mediated nick-end labeling (TUNEL) staining

The tissue sections were deparaffinated in xylene and rehydrated in descending concentrations of ethanol, followed by antigen retrieval in sodium citrate buffer for 10 min at room temperature and in a microwave oven at 100°C for 15 min. Endogenous peroxidase was inhibited by incubation with 3% hydrogen peroxide for 10 min at room temperature. Then tissue sections were stained by TUNEL using the ApopTag kit (Roche, USA) according to the manufacturer’s protocol. The sections were then stained with hematoxylin. The apoptotic index was calculated as the percentage of TUNEL-positive cells in granulosa cells.

### 2.9 Quantitative real-time PCR analysis

RNA was extracted from the ovary samples using RNAiso Plus (Takara Bio Inc.) according to the manufacturer’s instructions. The RNA samples were then reverse-transcribed into cDNA using the PrimerScript RT Master Mix Perfect Real-Time Kit (Takara Bio Inc.). The PCR primers were designed using primer premier 5.0 software. Table 1 presents a list of the PCR primers for *GAPDH*, *SOD1*, *SOD2*, *GPX1*, *GPX2*, *STAR*, *3β-HSD*, *17β-HSD I*, *17β-HSD II*, *P450scc*, and *P450arom*. The PCR reaction system consisted of 2 μL cDNA, 10 μL of 2× SYBR Ex *Taq* primer (DRR041S; Takara Bio Inc.), 0.6 μL each of sense and antisense primer, and 6.8 μL of double-distilled water, according to the manufacturer’s protocol. Quantitative PCR was performed using the Bio-Rad IQ5 qPCR thermocycler (Bio-Rad, Berkekey, CA). The target gene mRNA expression levels were normalized to the GAPDH expression using the following formula:

$$\text{Relative quantity of target gene mRNA}=2^{-\Delta \Delta \text{Ct}}$$

$$\begin{array}{l} △△\text{Ct}=({\text{Ct}}_{\text{target gene mRNA}}\;-\;{\text{Ct}}_{\text{GAPDH mRNA}}\;)\;\text{test group}-\\ ({\text{Ct}}_{\text{target gene mRNA}}-{\text{Ct}}_{\text{GAPDH mRNA}}\;)\text{control group} \end{array}$$

**Table 1:** Primer sequences used in the study for real-time PCR.

Gene	Accession number	Primer sequence (5’→3’)	Expected PCR products size
*SOD1*	NM_017050.1	Sense: CTTCTGTCGTCTCCTTGCTT, Anti-sense: TCTGCTCGAAGTGAATGACG	136
*SOD2*	NM_017051.2	Sense: CTGGCCAAGGGAGATGTTAC, Anti-sense: CAGCAACTCTCCTTTGGGTT	138
*GPX1*	NM_030826.3	Sense: TCAGTTCGGACATCAGGAGA, Anti-sense: GAAGGTAAAGAGCGGGTGAG	148
*GPX2*	NM_183403.2	Sense: CAACATCGAGCCTGACATCA, Anti-sense: AAATGCTTCGGTTTCCCAGT	145
*StAR*	NM_031558.3	Sense: CCCAAATGTCAAGGAAATCA, Anti-sense: TCAGGCATCTCCCCAAAGTG	189
*P450scc*	NM_017286.2	Sense: CTGCCTGGGATGTGATTTTC, Anti-sense: CAGAGTCATGGAGGTCGTGT	200
*3β-HSD*	XM_003749361.2	Sense: ACTGGCTTGCCTTCCTGC, Anti-sense: CTGAGCGAGCGGAAGAAGATGC	180
*17β-HSDI*	NM_010475.1	Sense: GGTTATGAGCAAGCCCTGAG, Anti-sense: GGAAGCGGTTTGTGGAGAA	115
*17β-HSDII*	NM_024391.1	Sense: AGGTGTTTCTGCCTCTACTT, Anti-sense: CTGTGAGCCTACGATGTTT	218
*P450arom*	NM_017085.2	Sense: GCCTGTCGTGGACTTGGT, Anti-sense: TAAATTCATTGGGCTTGG	140
*GAPDH*	NM_017008.4	Sense: AACGACCCCTTCATTGACCTC, Anti-sense: CCTTGACTGTGCCGTTGAACT	85

### 2.10 Statistical analysis

The differences between the treatment and control groups were statistically analyzed by one-way analysis of variance (ANOVA). Multiple comparison was carried out using the least-significant differences (LSD) test. All statistical analyses were carried out using the Statistical Package for Social Sciences (SPSS version 20.0 for Windows). Results were presented as the means ± standard deviation (SD). P values of 0.05 or 0.01 were considered statistically significant and labeled with an asterisk (*) or (**) on each graph.

## Results

### 3.1 Effect of cyanuric acid on the female rats

**Fig 1:**
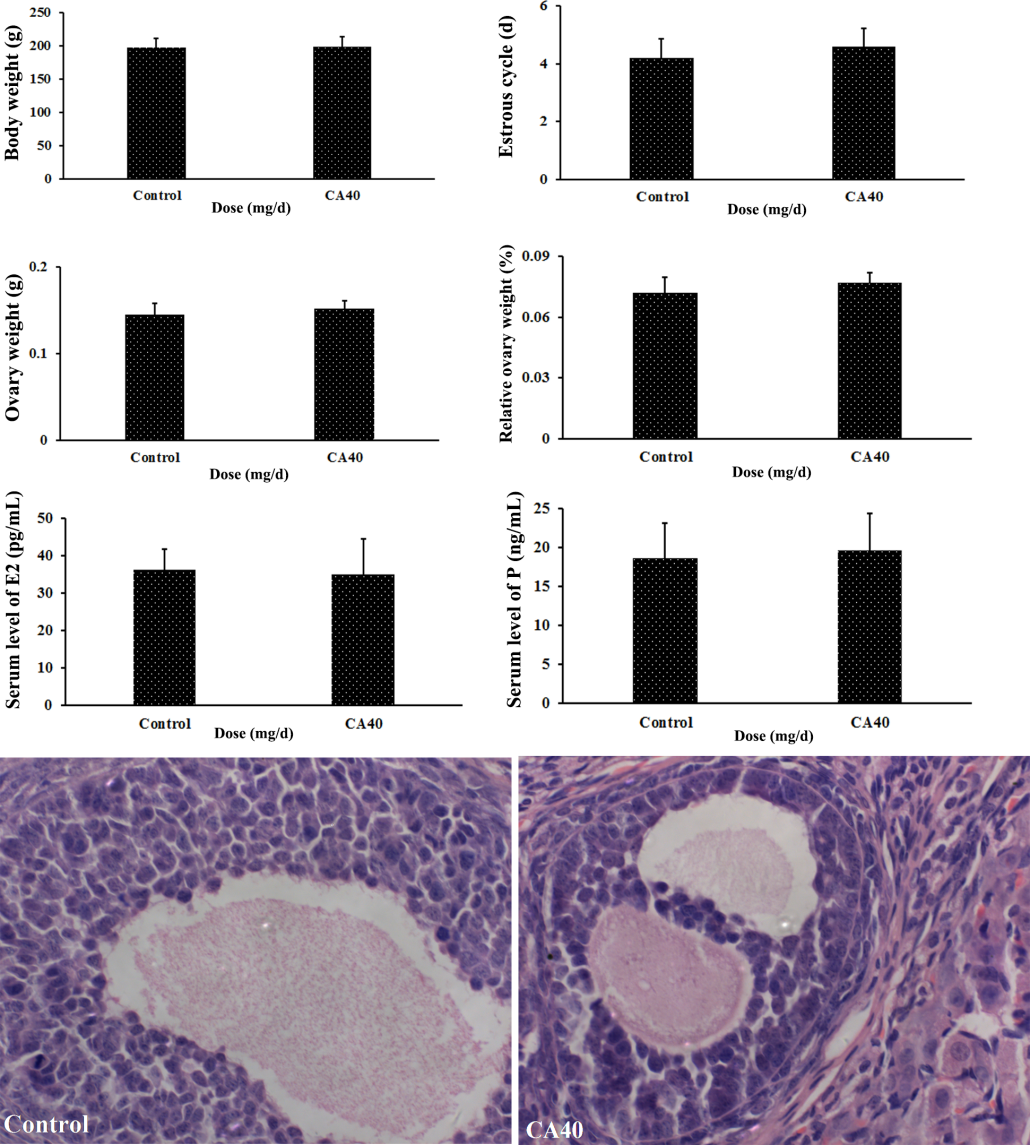
Effect of cyanuric acid (CA) on female rats. Values are expressed as means ± SD, n = 5. *P < 0.05, **P < 0.01: CA group versus control group. Ovarian sections are stained with hematoxylin and eosin.

To determine the toxicity of CA to the female rats, the body and ovarian weights, estrous cycle, serum levels of E2 and P and ovarian histopathological change were observed after exposure to 40 mg·(kg·d)^–1^ CA. No significant difference was found in body and ovarian weights, duration of the estrous cycle and levels of E2 and P in CA-treated group and the control group (P > 0.05), and no obvious morphological changes in the ovaries was observed in CA-treated group (Fig 1). These results indicate that CA has no toxicity to the ovary. Hence, in the next study we only investigated the toxicities of MA and MC to female rats.

### 3.2 Effect of melamine and the melamine-cyanuric acid mixture on the general health of female rats

**Fig 2:**
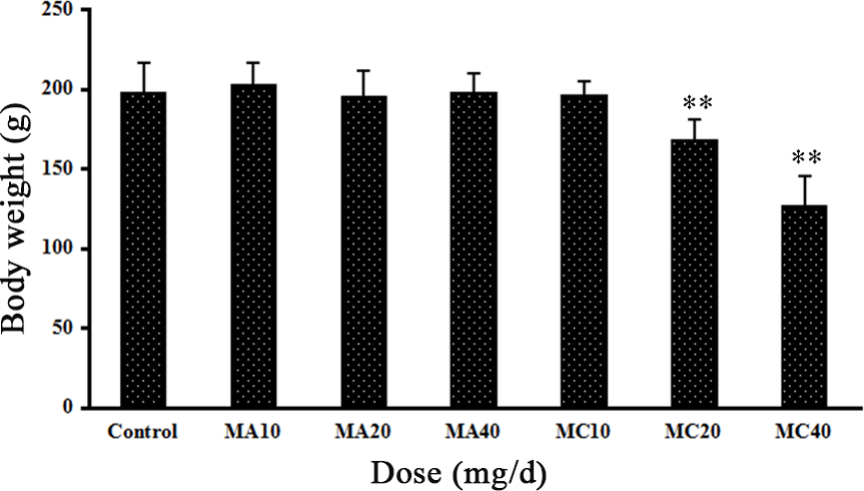
Body weights of rats after exposure to different doses of melamine (MA) and melamine and cyanuric acid (MC). Values are expressed as means ± SD, n = 5. *P < 0.05, **P < 0.01: MA group versus control group and MC group versus control group.

To determine the effects of melamine on the general health of female rats, we observed the body weight, appetite, and other behavioral and morphological parameters after exposure to chemicals and compared them with the control (no exposure) group. The body weights steadily increased in all three MA groups and there were no statistically significant differences between the three groups. However, rats in the MC group had rough hair and showed reduced food and water intake (20 and 40 mg·(kg·d)^–1^), and their physical condition worsened and their food and water intake gradually decreased over time during the experimental period. The body weights of these rats significantly decreased by 15.3% and 37.4% reduction in the 20 and 40 mg·(kg·d)^–1^ MC groups after 28 days as compared with the controls (P < 0.01). Body weights in the MA groups did not differ significantly from those in the control group (Fig 2). None of the rats died at any time during the experiment.

**Fig 3:**
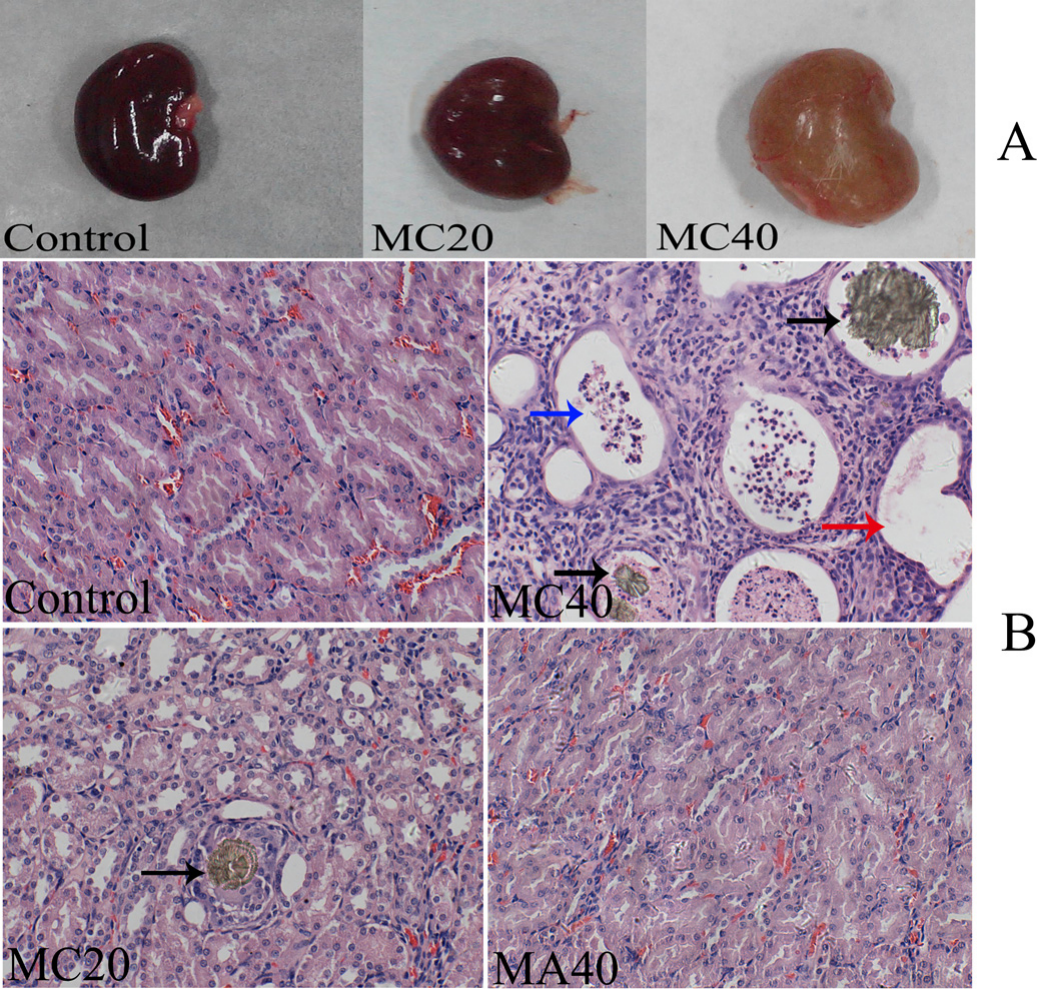
Gross pathological and histopathological changes of kidneys. Hematoxylin-eosin staining illustrating the gold-brown crystals (black arrow), cellular casts (blue arrow) and compensatory expansion (red arrow).

**Fig 4:**
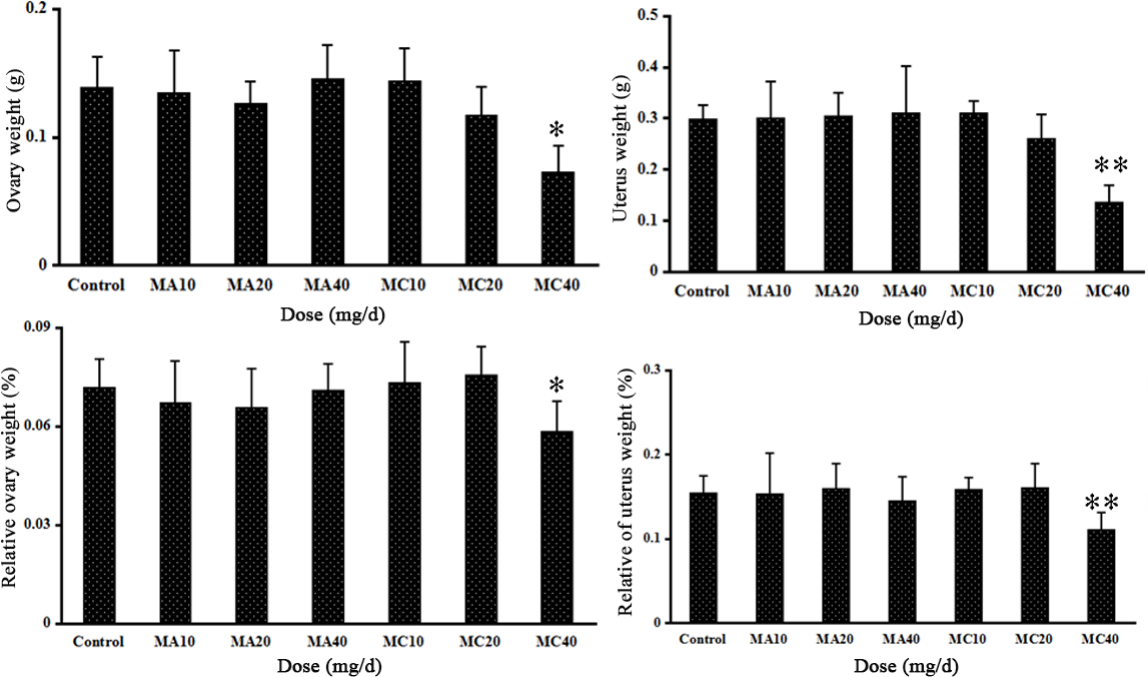
Absolute and relative ovary and uterus weights of rats exposed to different doses of melamine (MA) and melamine and cyanuric acid (MC). Values are expressed as means ± SD, n = 5. *P < 0.05, **P < 0.01: MA group versus control group and MC group versus control group.

Investigation of the organs after euthanasia revealed kidney enlargement with gold-brown colors in the 20 and 40 mg·(kg·d)^–1^ MC groups, and these changes were considerably more severe in the 40 mg·(kg·d)^–1^ MC group (Fig 3A). There were no obvious gross changes in the kidneys of any of the other treatment groups (Fig 3A). The absolute and relative ovarian and uterine weights were significantly lower in the highest concentration MC group (40 mg·(kg·d)^–1^) than in the control group (P < 0.05), and no changes were found in the other groups (Fig 4).

### 3.3 Effect of MA and MC on the estrous cycle

**Fig 5:**
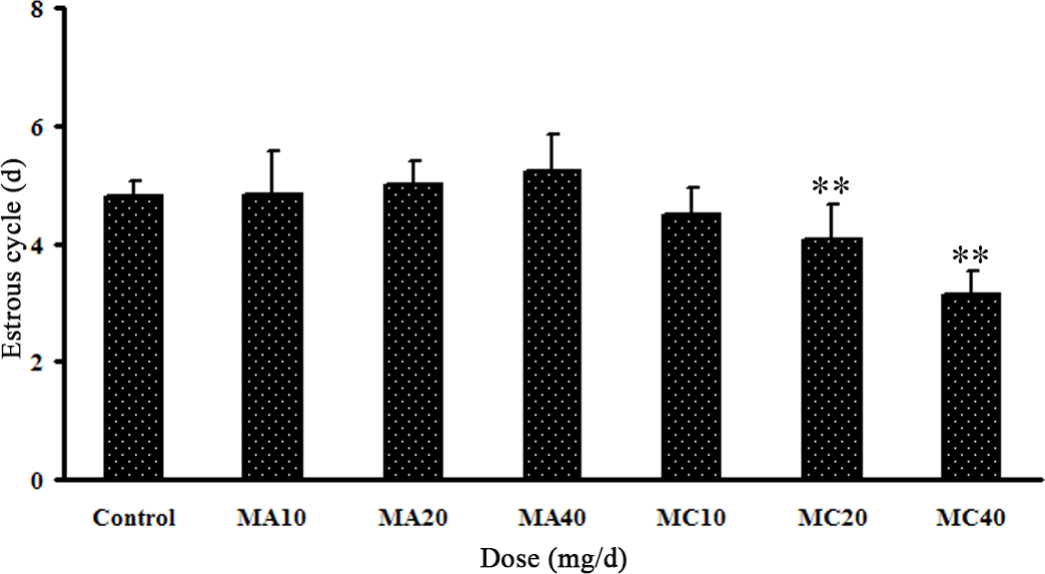
Estrous cycles of rats exposed to different doses of melamine (MA) and melamine and cyanuric acid (MC). Values are expressed as means ± SD, n = 5. *P < 0.05, **P < 0.01: MA group versus control group and MC group versus control group.

The estrous cycle of the control group rats lasted about 4–6 days, while the estrous cycles of the MC rats were shorter. The duration of the estrous cycle was significantly lower in the 20 and 40 mg·(kg·d)^–1^ MC groups than in the control group (P < 0.05), and there were no differences in other groups (Fig 5).

### 3.4 Effects of MA and MC on serum E_2_ and P concentrations

**Fig 6:**
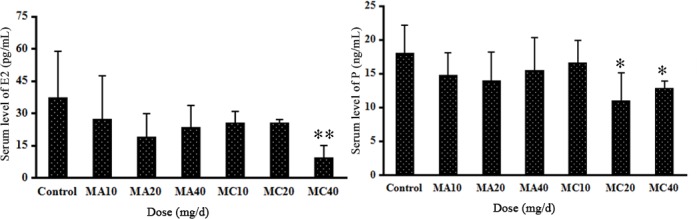
Serum levels of estrogen and progesterone of rats exposed to different doses of melamine (MA) and melamine and cyanuric acid (MC). Values are expressed as means ± SD, n = 5. *P < 0.05, **P < 0.01: MA group versus control group and MC group versus control group.

The serum E_2_ and P levels are shown in Fig 6. The serum E_2_ and P levels decreased in a dose-dependent manner after exposure to MA or MC, and their concentrations were lower in the MC groups than in the MA groups. Furthermore, there was a significant decrease in the E_2_ level in the 40 mg·(kg·d)^–1^ MC group (P < 0.01), and the serum P level was significantly lower in the 20 and 40 mg·(kg·d)^–1^ MC groups compared with the control (P < 0.05). No statistically significant differences were found in the other groups.

### 3.5 Effects of MA and MC on kidney and ovarian morphology and atretic follicles

**Fig 7:**
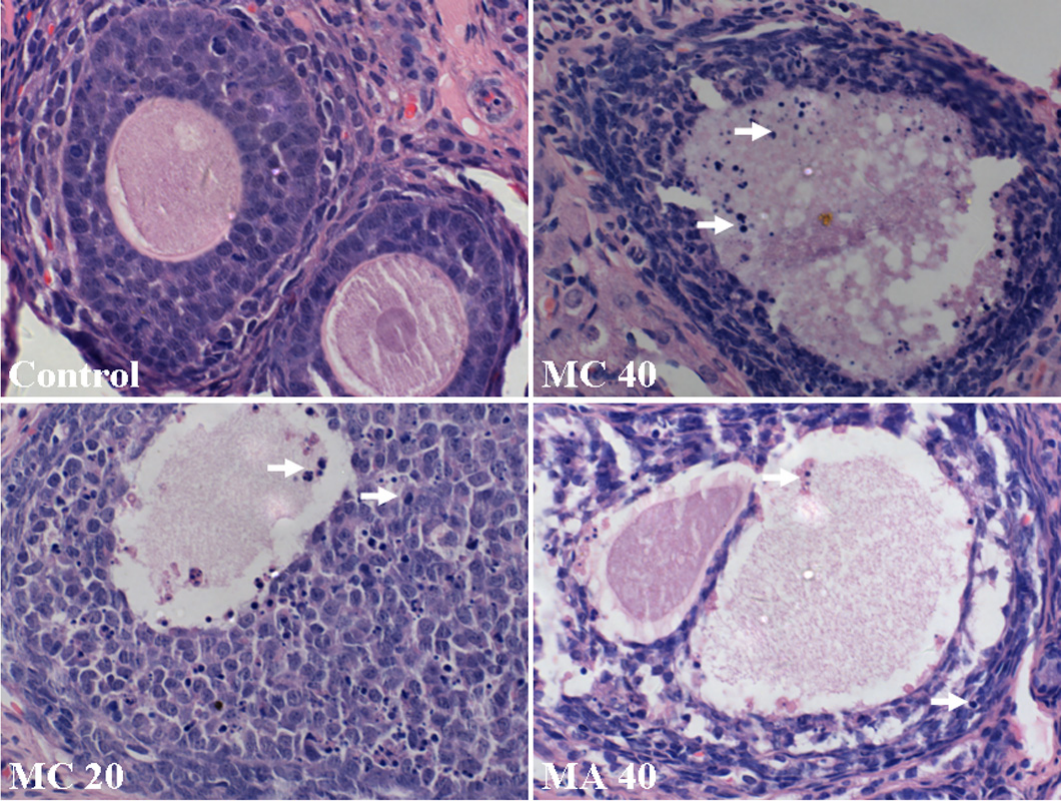
Photomicrographs of ovary sections. Hematoxylin-eosin staining illustrating the necrosis of granulosa cells (white arrows).

**Fig 8:**
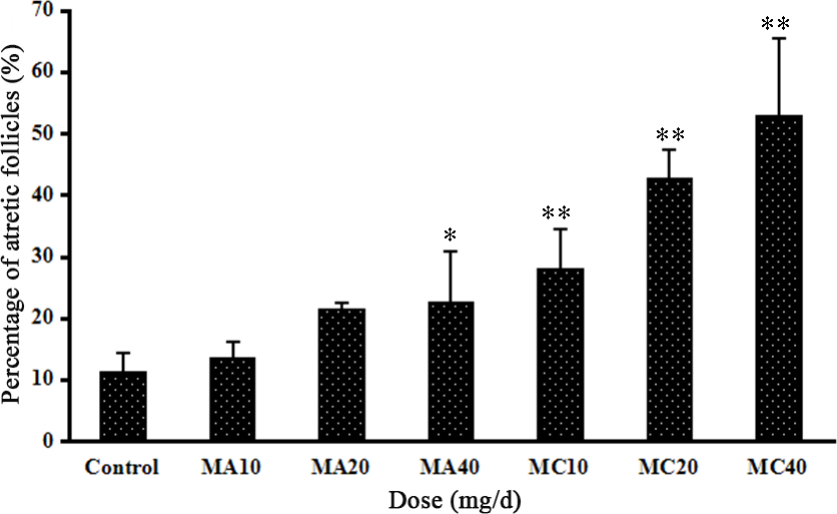
Numbers of atretic follicles in ovaries after rats were treated with different doses of melamine (MA) and melamine and cyanuric acid (MC). Values are expressed as means ± SD, n = 5. *P < 0.05, **P < 0.01: MA group versus control group and MC group versus control group.

No toxic lesions were observed in the control, MA-treated groups and 10 mg·(kg·d)^–1^ MC group. However, slight fan-shaped, gold-brown crystals were found in the distal tubules in the 20 mg·(kg·d)^–1^ MC group (Fig 3B). Crystals were also found in the 40 mg·(kg·d)^–1^ MC group, with more crystals in the distal tubules than in the collecting ducts. In addition, cellular casts and compensatory expansion were found in the distal tubules (Fig 3B). Both MA and MC also induced obvious morphological changes in the ovaries in a dose-dependent manner, and MC induced more severe ovarian lesions than MA. The morphological changes were mainly observed in the follicles. The ovaries of control rats exhibited normal architecture, with follicles at different stages of development with oocytes exhibiting distinct nuclei and aligned granulosa layers. However, necrosis of oocyte nucleus and granulosa cells, thin layers of granulosa cells, and detachment of granulosa cells from the basement membrane were observed in the groups exposed to 40 mg·(kg·d)^–1^ MA and all the MC groups (Fig 7). Moreover, the exposure groups had higher numbers of atretic follicles, and the 40 mg·(kg·d)^–1^ MA group and the MC groups showed a significantly higher number of atretic follicles compared to the control group (P < 0.05, Fig 8).

### 3.6 Effect of MA and MC exposure on apoptosis of granulosa cells

**Fig 9:**
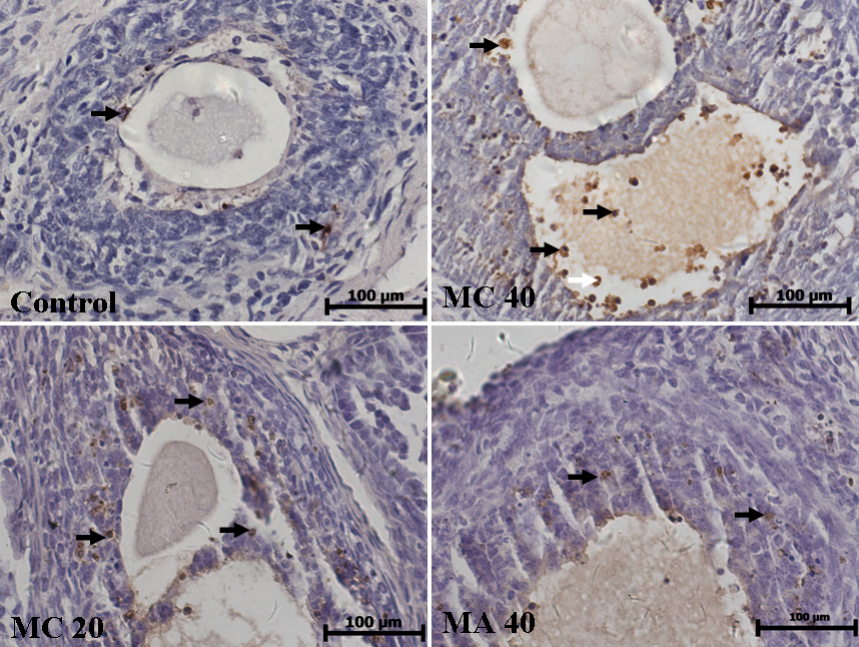
Apoptotic cells in rat ovaries detected by TUNEL staining after the rats were exposed to different doses of melamine (MA) and melamine and cyanuric acid (MC). TUNEL-positive cells were found in the granulosa cells (black arrows).

**Fig 10:**
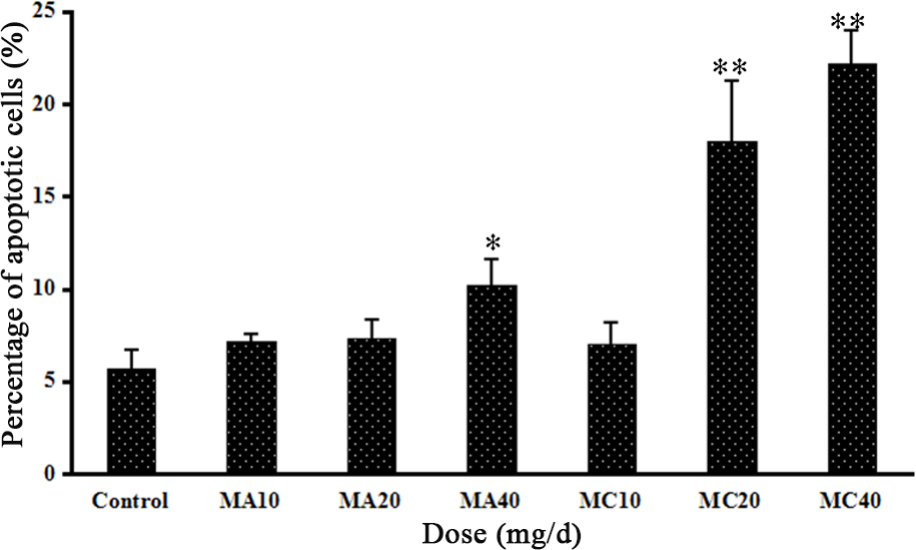
Percentages of apoptotic cells in ovaries after rats were exposed to different doses of melamine (MA) and melamine and cyanuric acid (MC). Values are expressed as means ± SD, n = 5. *P < 0.05, **P < 0.01: MA group versus control group and MC group versus control group.

TUNEL staining was used to detect apoptosis in granulosa cells in rats of all the three groups (Fig 9). TUNEL-positive cells were found in the granulosa cells, and the number of TUNEL-positive cells increased with increase in the MA and MC doses. The percentages of apoptotic cells in the 40 mg·(kg·d)^–1^ MA group and the 20 and 40 mg·(kg·d)^–1^ MC groups were significantly higher than the percentage of apoptotic cells in the control group (P < 0.05; Fig 10). This finding is consistent with the observed morphological changes of granulosa cells.

### 3.7 Effects of MA and MC on antioxidant enzymes

**Fig 11:**
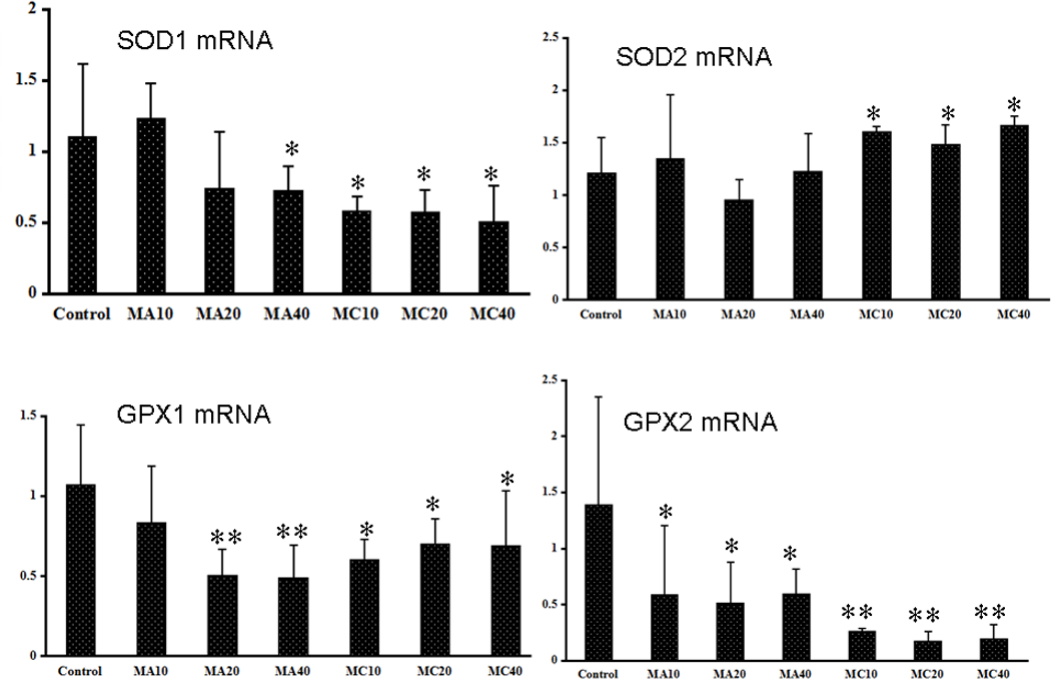
mRNA expressions of antioxidant enzymes in the ovaries of rats exposed to melamine (MA) and melamine and cyanuric acid (MC). Values are expressed as means ± SD, n = 5. *P < 0.05, **P < 0.01: MA group versus control group and MC group versus control group.

Fig 11 presents the *SOD1*, *SOD2*, *GPX1*, and *GPX2* mRNA levels in all the groups. The *SOD1* mRNA level was significantly lower in the 40 mg·(kg·d)^–1^ and other MC groups compared to the control group (P < 0.05), while the *SOD2* mRNA levels were obviously higher in the MC groups than in the controls (P < 0.05). The *GPX1* mRNA levels in the 20 and 40 mg·(kg·d)^–1^ MA and MC groups and the *GPX2* mRNA level in the MC group were significantly lower than the corresponding levels in the control group (P < 0.05).

### 3.8 Effects of MA and MC on genes associated with steroidogenesis

**Fig 12:**
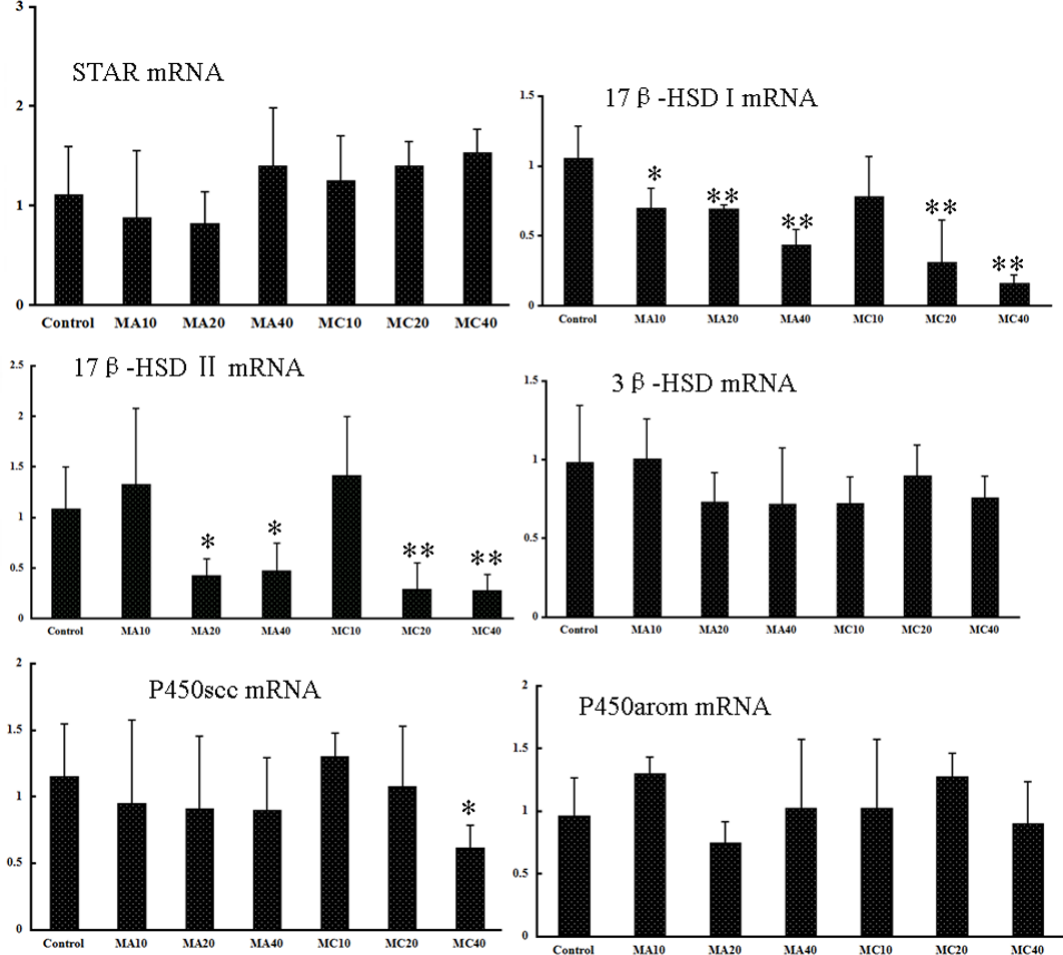
mRNA expression levels of genes associated with steroidogenesis in ovaries of rats exposed to melamine (MA) and melamine and cyanuric acid (MC). Values are expressed as means ± SD, n = 5. *P < 0.05, **P < 0.01: MA group versus control group and MC group versus control group.

Fig 12 lists the *STAR*, *17β-HSD I*, *17β-HSD II*, *3β-HSD*, *P450scc*, and *P450arom* mRNA levels in all the groups. The *17β-HSDI* and *17β-HSDII* mRNA levels in the 20 and 40 mg·(kg·d)^–1^ MA and MC groups were significantly lower than the corresponding values in the control group (P < 0.05). The *P450scc* mRNA level in the 40 mg·(kg·d)^–1^ MC group was significantly lower than the corresponding mRNA levels in the control group (P < 0.05). No differences were found in the mRNA expression levels of *STAR*, *3β-HSD*, and *P450arom* in any of the groups.

## Discussion

It is reported that cyanuric acid is practically nontoxic when administered as a single oral or dermal dose. According to a previous study, the oral LD50 of cyanuric acid in rats is greater than 10,000 mg/kg [9–10]. More than 98% of the dose of cyanuric acid was recovered unchanged in urine after 24 h and the excretion half-life time is approximately 3h [9,11]. Moreover, recent studies have shown that cyanuric acid alone at 30 mg·(kg·d)^–1^ treatment for 7 days does not cause damage to rats and has no effect on dam and fetal growth if given to pregnant rats even when given very high doses (1000 mg·(kg·d)^–1^) [20–21]. In our preliminary experiment, no toxicity was found in female rats after treatment with 40 mg·(kg·d)^–1^ CA. Against this background, in this study we only investigated the toxicities of MA and MC to female rats. In addition, it has been reported that 10.6 mg·(kg·d)^–1^ MC (5.3mg·(kg·d)^–1^ melamine + 5.3mg·(kg·d)^–1^ cyanuric acid) is nontoxic based on histopathological studies of kidney in rats treated for 28 days [22]. Against this background, 10 mg·(kg·d)^–1^ MA or MC were chosen as a low doses and 20 and 40 mg·(kg·d)^–1^ MA or MC were chosen as moderate and high doses, respectively, as previous studies had demonstrated that these are associated with obvious renal toxicity.

Numerous studies have reported the toxicities of MA and MC on the kidneys and testes, but little is known about their possible toxicities on the female reproductive system. To the best of our knowledge, this is the first study to determine the damage caused by MA and MC to rat ovaries. MA had no effect on general heath compared to controls, but ovarian lesions, atretic follicles, apoptosis of granulosa cells and a reduction of antioxidant enzymes were observed in MA groups, indicating that MA is specifically toxic to female reproductive functions in rats. This is consistent with a recent report that MA causes a reduction of oocyte quantity and fertility including abnormal oocyte cytoskeletons, abnormal mitochondrial distributions, and early stage apoptosis/autophagy in mice after 8 weeks of treatment [23–24]. Rats that were exposed to MC had rough hair; reduced food and water intake; decreased body, ovary and uterus weights; and severe ovarian lesions; indicating that MC was considerably more toxic than melamine alone. Renal crystals were found in the MC-treated rats, but MA-treated rats had no renal crystals, demonstrating that the more severe effects observed following MC exposure may be related to the formation of melamine-cyanuric acid crystals in the kidneys. In addition, no body weight loss was observed in the low MC group, while atretic follicles and a reduction of antioxidant enzymes were found, indicating that the ovarian lesions were caused by the MC itself, rather than the loss of body weight. However, significant weight loss and more severe ovarian lesions were found in the middle and high dose MC groups, demonstrating that both MC toxicity and weight loss may contribute to the ovarian lesions. From this, we can also infer that the damages to the kidneys and ovaries leads to a reduction in food intake, and then finally to body weight loss.

The MA and MC-treated rats were found to have atretic follicles and granulosa cell lesions, while luteal cells and connective tissues showed no obvious morphological changes, indicating that these compounds mainly caused damage to follicles. The damage caused by MA and MC to follicles may be related to the sensitivity of follicles to external compounds, which has been previously reported in other studies [18–19,25]. In addition, several previous studies have reported that melamine can pass through the blood-testis barriers in male mice and then cause lesions to the testes, and melamine has been found in the amniotic and breast fluid in female rats [15,17,26–27]. Taken together, these results suggest that melamine can enter the ovaries and damage them. In this study, MC induced more severe lesions than MA, demonstrating that cyanuric acid plays an important part in the formation of these lesions. However, previous studies have reported that cyanuric acid alone is nontoxic and does not cause damage to kidneys or testes [9, 22], and also no ovarian lesion was found in our preliminary study. Hence, the more severe lesions may be a consequence of the crystals formed by melamine and cyanuric acid. Previous studies have shown that melamine and cyanuric acid can combine to form crystals in the kidneys and testes and then cause lesions [11,15,27], which suggest that melamine is highly toxic in combination with cyanuric acid. No ovarian crystals were found in this study. This might due to the low doses of melamine and cyanuric acid used or very few crystals may have been formed but not retained in the ovaries. Previous studies have demonstrated severe renal and testicular lesions induced by MC even when crystals could not be microscopically observed [13,15,28]. However, MC-treated rats showed a reduction in the body weight and had yellow-brown crystals in the kidneys. Accordingly, secondary anorexia and renal failure should be taken into account for ovarian lesions.

To investigate the mechanism by which these compounds induce lesions, we stained the cells by TUNEL to measure apoptosis and determined the levels of oxidative stress. The MA- and MC-treated rats were found to have TUNEL-positive granulosa cells; these findings correlated well with the morphological changes observed under the microscope. Rats exposed to MA and MC had significantly higher numbers of apoptotic cells compared to the control rats, indicating that apoptosis might contribute to the lesion of ovaries. The *SOD1*, *GPX1*, and *GPX2* mRNA expression levels in the MA and MC groups were obviously lower than the corresponding levels in the control, demonstrating that the balance between reactive oxygen species (ROS) production and the antioxidative activity of enzymes are destroyed following MA and MC treatment and large amounts of ROS accumulate in ovaries that may consequently damage the ovaries. Oxidative stress induced by melamine has been previously reported as a factor contributing to testis damage [13]. Moreover, oxidative stress can induce apoptosis by the mitochondrial pathway [29–30]. Similarly, the apoptosis of granulosa cells observed in the MA and MC groups in this study may be related to the oxidative damage. Previous studies have reported that melamine-induced apoptosis in NREK-52e cells is partly caused by oxidative stress [31–32]. However, it is unclear why the SOD2 mRNA expression is increased after MA and MC exposure, and further studies are required to clarify this.

E_2_ plays important roles in the estrous cycle, ranging from growth of primordial follicles to ovulation in each cycle [33–34]. Abnormal antral follicles with uneven granulosa cell layers have been previously found in P450-null mice unable to produce E_2_ [16]. The serum E_2_ level in the treated rats in this study obviously decreased, especially following treatment with MC. The reduced E_2_ level may be why the estrous cycle duration is reduced and the number of atretic follicles is increased after exposure to MA and MC. Cholesterol is required for the production of E_2_ and P. StAR transfers cholesterol from the outer membrane to the inner mitochondrial membrane, where the P450scc enzyme is located. The cholesterol is converted into pregnenolone by P450scc, and then metabolized to P by 3β-HSD [35–36]. 17β-HSD and P450arom are crucial for E_2_ synthesis. In this study, the *P450scc*, *17β-HSDI*, and *17β-HSDII* mRNA expression levels decreased while the *StAR*, *3β-HSD*, and *P450arom* mRNA expression levels showed no change, indicating that the decreased serum P and E_2_ levels are caused by the reduced *P450scc* and *17β-HSD* levels. Indeed, the necrosis of granulosa cells is also involved in the reduced E_2_ levels because they are the main source of E_2_. In addition, as E_2_ is known to inhibit the apoptosis of granulosa cells [34], the apoptotic cells observed in this study could be attributed to the decreased levels of E2.

In conclusion, the MC mixture resulted in a reduction in the ovarian and uterine weights, shorter estrous cycles, and serum levels of P and E_2_ in female rats, and MA and MC both caused ovarian lesions, including increased numbers of atretic follicles and necrosis of granulosa cells and oocytes. Moreover, ovaries of MA- and MC-treated rats showed apoptosis of granulosa cells, oxidative damage, and decreased expressions of genes associated with steroidogenesis, indicating that both MA and MC are toxic to rat ovaries. The apoptosis and oxidative stress contributed to ovarian lesions, and the decreased activities of steroidogenic enzymes consequently decreased the serum E_2_ and P levels. It is noteworthy that MC caused more severe damage to the ovaries than MA.
